# Moving to an “Active” Biophilic Designed Office Workplace: A Pilot Study about the Effects on Sitting Time and Sitting Habits of Office-Based Workers

**DOI:** 10.3390/ijerph16091559

**Published:** 2019-05-04

**Authors:** Birgit Wallmann-Sperlich, Sophie Hoffmann, Anne Salditt, Tanja Bipp, Ingo Froboese

**Affiliations:** 1 Institute for Sports Science, Julius-Maximilian University Würzburg, 97082 Würzburg, Germany; sophie.hoffmann@stud-mail.uni-wuerzburg.de; 2 Interface Deutschland GmbH, Krefeld, 47803 Krefeld, Germany; Anne.Salditt@interface.com; 3 Work, Industrial, and Organizational Psychology, Julius Maximilian University of Würzburg, 97070 Würzburg, Germany; tanja.bipp@psychologie.uni-wuerzburg.de; 4 Institute of Health Promotion and Clinical Movement Science, German Sport University Cologne, 50933 Cologne, Germany; froboese@dshs-koeln.de

**Keywords:** desk-based, office-workers, standing, online survey, walking, work engagement, habit strength, work performance, office environment

## Abstract

Promising initial insights show that offices designed to permit physical activity (PA) may reduce workplace sitting time. Biophilic approaches are intended to introduce natural surroundings into the workplace, and preliminary data show positive effects on stress reduction and elevated productivity within the workplace. The primary aim of this pilot study was to analyze changes in workplace sitting time and self-reported habit strength concerning uninterrupted sitting and PA during work, when relocating from a traditional office setting to “active” biophilic-designed surroundings. The secondary aim was to assess possible changes in work-associated factors such as satisfaction with the office environment, work engagement, and work performance, among office staff. In a pre-post designed field study, we collected data through an online survey on health behavior at work. Twelve participants completed the survey before (one-month pre-relocation, T_1_) and twice after the office relocation (three months (T_2_) and seven months post-relocation (T_3_)). Standing time per day during office hours increased from T_1_ to T_3_ by about 40 min per day (*p* < 0.01). Other outcomes remained unaltered. The results suggest that changing office surroundings to an active-permissive biophilic design increased standing time during working hours. Future larger-scale controlled studies are warranted to investigate the influence of office design on sitting time and work-associated factors during working hours in depth.

## 1. Introduction

Most employees spend the majority of their waking hours at work. Over recent decades, work has shifted away from physically demanding tasks towards more sedentary tasks, resulting in a decreased contribution of occupational physical activity (PA) to overall PA [1,2] and substantially increasing sitting time. Sedentary behavior, primarily sitting, is associated with various negative health outcomes, including all-cause mortality, cardiovascular diseases, and diabetes, independently of physical inactivity [3,4,5]. Occupational sitting time is one of the most prominent contributors to overall daily sitting time in white-collar workers [6,7,8], who are particularly exposed to the health risks of prolonged sitting [9,10,11]. Current recommendations to counteract office-induced prolonged sitting are based mainly on expert consensus [12] and advise office workers to reduce sitting time during office hours by up to 50% and interrupt prolonged sitting every 20–30 min with standing and moving [13] (see also https://beupstanding.com.au/theevidence/). Consequently, there is a growing public health interest in the office-based setting to develop solutions, allowing for the reduction and interruption of prolonged sitting time and consequently providing possibilities for micro-bouts of PA. Strategies within the workplace to reduce and interrupt sitting time include information and counseling for reducing sitting time during office hours [14], as well as physical workplace modifications, such as replacing sitting with standing through combined sit and stand desks [15,16,17]. However, a Cochrane review concluded that evidence that sit-and-stand desks reduce sitting time at work was of low quality [18]. Further systematic reviews and meta-analyses of workplace intervention strategies argue that the combination of educational/behavioral and environmental interventions, referred to as multicomponent interventions, are the most promising for reducing sitting time [18,19,20].

Early evidence also suggests that the design of the workplace building can influence the office-worker’s cognitive, social, psychological and physical health [21,22] by incorporating special design approaches such as biophilic [21,23] or active design [24]. In this regard, the primary focus of the biophilic design is to introduce nature into the built environment, inducing lower levels of stress [21,25], and improving workplace satisfaction [26] and productivity [21,26]. Approaches in the office-based setting include open plan workspaces, natural lighting, ventilation, plants, views, and the use of recycled and non-synthetic materials [21].

“Active design” is a relatively new concept in design, integrating building and planning principles to promote PA and reduce sitting time. The multi-disciplinary active design approach seeks to translate evidence-based research into practical design solutions and addresses features of the built environment to ensure support for daily PA and to reduce workers’ sitting time. Two Australian short-term experiments showed that relocating to an “activity permissive” building and to a building exhibiting active design, respectively, promoted less sitting and more standing in desk-based workers [27,28,29]. These results regarding reduced sitting time warrant further evaluation, especially concerning their sustainability and to gain deeper insights regarding the effects of the different health-enhancing features induced by the active design.

To some extent, habit seems to explain sedentary behavior in the office setting [30,31], as workplace sitting seems to be an unconscious behavior for most workers that accompanies work tasks and the characteristics of the office environment [32]. Office modifications such as standing meeting tables, task-specific workstations, sit-and-stand workstations, etc., could potentially promote habit-breaking attempts, thereby breaking up and reducing sitting time [30,33,34]. Therefore, research related to investigating the influence of active design on breaking the habit of prolonged sitting and targeting PA behavior in office-based settings is warranted.

Furthermore, along with alterations to the workplace environment, motivational factors, such as satisfaction with the office environment, work engagement—characterized by a positive, fulfilling, state of mind regarding work [35], and work performance also have to be considered. Although positive effects on employees’ motivation are reported for modern, innovative office concepts [36], studies also point towards the potential negative effects—for example, of open office concepts—on the health status or job satisfaction of employees [37]. Therefore, it is also important to evaluate the possible effects of active biophilic office designs on work engagement and work performance.

Hence, the overall aim of this pilot study was to analyze whether the relocation of a small-size office to an office building with an active biophilic design might alter workplace sitting time during working hours and self-reported habit strength concerning uninterrupted sitting and PA during work. The second aim was to evaluate changes in satisfaction with the office environment and important motivational and work outcomes of the office staff (in terms of workplace engagement and workplace performance).

## 2. Materials and Methods

### 2.1. Study Design

We conducted a pilot field study on office relocation with a pre-post design. Therefore, we collected data on sitting time at work, and included work-related outcomes in terms of office satisfaction, work engagement and work performance through an online survey. Participants completed the survey before (time point T_1_, one-month pre-relocation, September 2016) and twice after the office relocation (T_2_, three and seven months after relocation, January and May 2017). All study procedures were approved by the ethical review board of the Sports Institute of the University of Würzburg.

### 2.2. Sample

Participants were employees of a small-sized German subsidiary of a global supplier of modular flooring with an office-based workplace. Their office was redesigned and relocated to a revitalized building that had been then built under the guidance of Erich Holthoff, a former student of the leading Bauhaus architect Ludwig Mies van der Rohe, in the urban district of Krefeld in Germany. All 23 workers (managers, officers, secretarial staff, etc.) were invited to participate in the online survey through an email from the research team forwarded by the management. Follow-up emails reminding employees to participate were sent twice for each time point. Participation was voluntary, and no incentives were provided for study participation. We collected data from *n* = 23 participants at T_1_ but were only able to analyze data from 12 employees (9 female, average age: 39 ± 10 years), who participated in all three-time measurements and could be assigned using their own personal code word. The baseline values for those participants included in the further analyses did not differ concerning sitting, standing, walking and performing physically demanding tasks, from those of the participants who dropped-out (*p* > 0.2).

### 2.3. Office Characteristics

**Figure 1:**
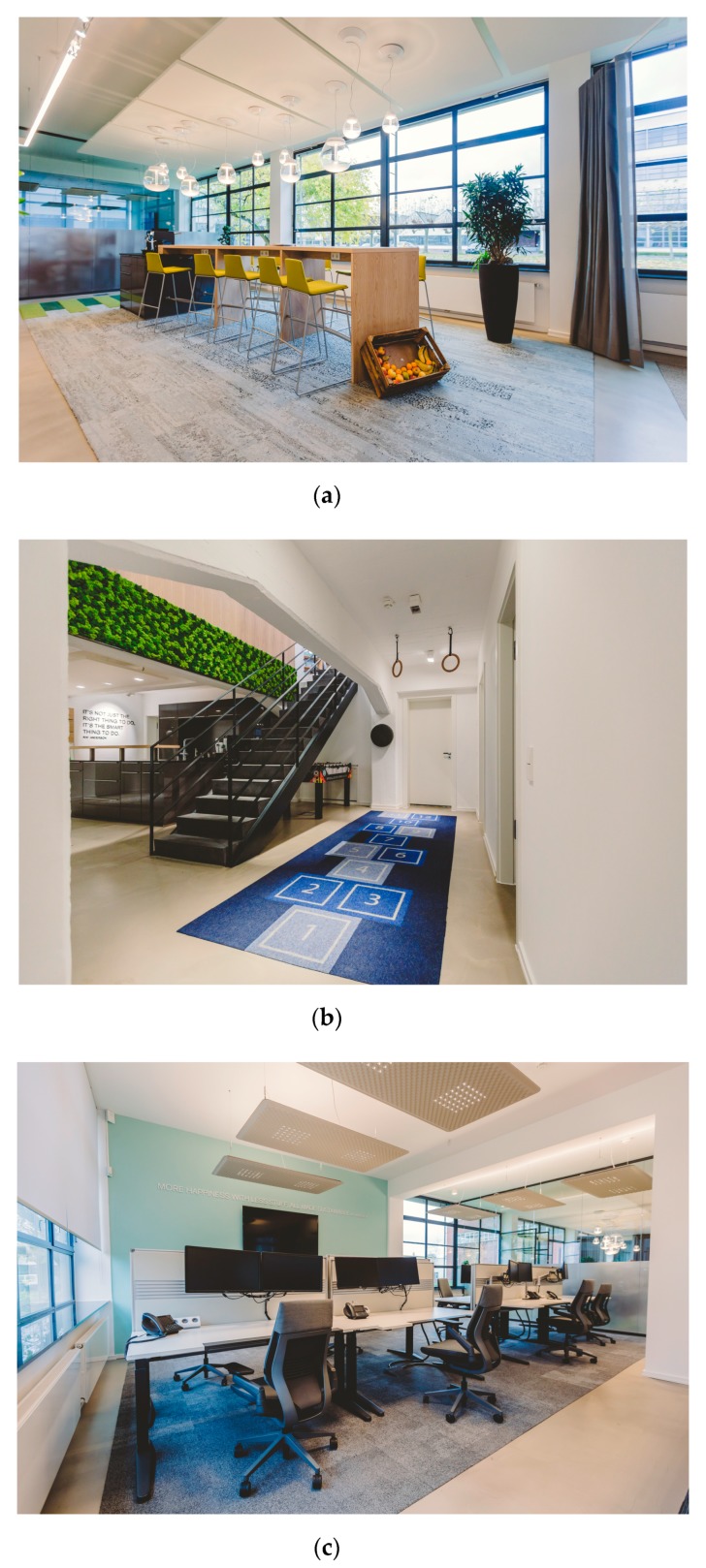
(**a**) New office—public space with counter and counter chairs (images: ©Lichthalle Krefeld); (**b**) ActiveOffice® features (image: ©Lichthalle Krefeld); (**c**) Employees’ work desks (image: ©Lichthalle Krefeld).

**Figure 2:**
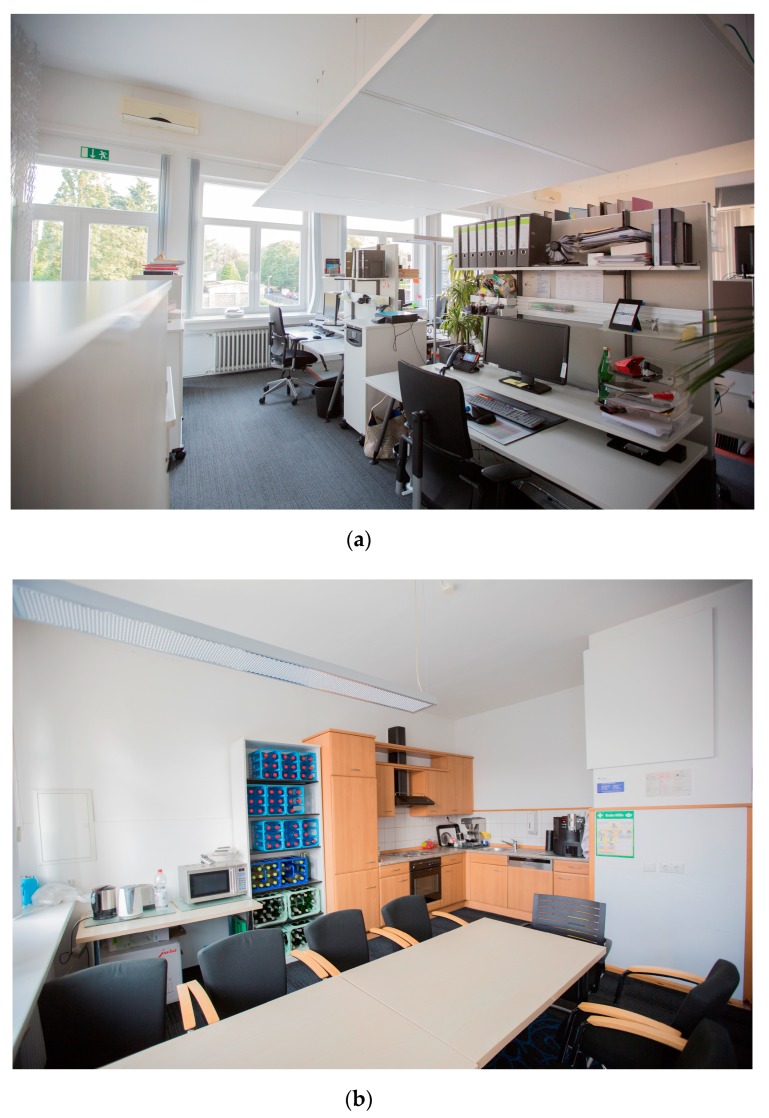
(**a**) Old office—employees’ work desks (image: ©Lichthalle Krefeld); (**b**) Old office—kitchen (image: ©Lichthalle Krefeld).

The new office design followed a biophilic design approach [23] with three dimensions: nature in the office, nature analogies, and the nature of the office. These included the use of open plan workspace, natural lighting, ventilation, significant numbers of plants, views, and recycled and non-synthetic materials. Furthermore, the office incorporated diverse possibilities for sitting and standing through height-adjustable workstations, a standing conference table with counter chairs, public spaces with counter tables and counter chairs, as well as different features permitting physical-activity (see Table 1 and Figure 1b). The characteristics of the old and new office are presented in Table 1, as well as some views of the old and new office in Figure 1a–c and Figure 2a,b.

### 2.4. Measures

#### 2.4.1. Total and Domain-Specific Sitting, Sitting Time and PA in the Workplace Setting 

To assess total and domain-specific sitting time during weekdays, we used the Marshall Sitting Questionnaire [38]. To further assess sitting time and PA in the office environment we adopted the Occupational Sitting and Physical Activity Questionnaire (OSPAQ) [39]. 

#### 2.4.2. Self-Reported Breaks in Sitting Time in the Workplace Setting

The following question, with acceptable properties concerning criterion validity [40], assessed the number of breaks in sitting time: ‘‘How many breaks from sitting (such as standing up, stretching, or taking a short walk) during one hour of sitting would you typically take at work?’’, a choice of responses (0, 1, 2, 3, 4, and 5 or more; I do not know) was given. 

#### 2.4.3. Availability and Use of Height-Adjustable Desks

All participants were asked if their office desk was electronically height-adjustable with the answer options, yes/no/don’t know. Participants who affirmed working at a height-adjustable desk were asked if they apply the height-adjustable function with the answer options, yes, regularly/irregularly/no. For further analyses, we merged the answer options into two groups “regular use” and “irregular/no use” as described in a prior study [41].

#### 2.4.4. Self-Reported Habit Strength Concerning Uninterrupted Sitting and PA during Work

Habit strength was assessed using the Self-Report Index of Habit Strength (SRIH) [42]. The SRHI comprises 12 items and measures the habit strength of a behavior by breaking it down into a number of features; i.e., history of repetition (e.g., “…I do frequently”), automaticity (lack of control, lack of awareness, efficiency) (e.g., “[Behavior X is something…] I have no need to think about doing”), and expression of one’s identity (“…that’s typically “me”). The behavior is specified by the introductory sentence ”Behavior X is something …”. Verplanken and Orbell [42] recommend a response scale anchored by agree–disagree, which should preferably contain five or more response categories.

Accordingly, we used a five-point Likert scale in this survey, and the participants were first asked to reflect on the behavior “uninterrupted sitting during work” and then on the behavior “physical activity during work”. For further analyses, we calculated the one-dimensional Index of Habit Strength concerning uninterrupted sitting and PA during work, where a high value reflects a high habit strength concerning the behavior. The SRHI revealed high internal and test-retest reliabilities [42].

#### 2.4.5. Satisfaction with the Office Environment

Participants were asked about their satisfaction with the office environment using the indoor environmental quality survey [43]. The instrument comprises office layout (four items), office furnishings (four items), thermal comfort (two items), indoor air quality (two items), lighting (three items), acoustics (three items), and overall satisfaction (one item). The response scale is a 7-point Likert scale anchored by “very satisfied” (1) and “very dissatisfied” (7). 

#### 2.4.6. Work Engagement

To assess work engagement, we used the shortened version of the Utrecht Work Engagement Scale–9 [44], which includes nine statements about how the participants feel at work. The participant must decide if he or she ever feels this way about his or her job on a scale of “never had this feeling” (0) to “always/every day have this feeling” (6). The positive work-related state of fulfillment is characterized by an overall score for work engagement with good internal consistency and test-retest reliability [44].

#### 2.4.7. Job Performance (Task and Contextual Performance)

We used the short scale of task and contextual performance originally introduced by Williams and Anderson [45] to measure job performance. The participants rated their opinion on a five-point Likert scale anchored by “disagree” (1) – “agree” (5) concerning four statements for: task performance (in role, required work duties, e.g., included in job description; i.e. “I adequately complete assigned duties”) and contextual performance (extra roles, such as extra/voluntary work behaviors that indirectly support the organization; i.e., “I help others who have heavy workloads”).

### 2.5. Data Analysis

For the item “availability and usage of height-adjustable desks” descriptive frequencies were calculated. For all other outcome variable repeated-measures ANOVA [time-point (pre-, 3-month post and 7-month post-relocation)] with Bonferroni correction was performed. To prevent inflation of type 1 error, we applied an alpha level of *p* < 0.01 as indicated by ∗. In addition, the values obtained were evaluated by calculating the effect size partial eta-squared (part η^2^). The means and standard deviations (SD) for all data sets were calculated and all statistical tests carried out in the SPSS 23.0 (IBM Corp., Armonk, NY, USA) software package for Microsoft. 

## 3. Results

All data collected from the twelve individuals who completed three-time points of the online survey are presented in Table 2. During baseline, participants reported a mean of 364.2 ± 102.5 min of sitting, 70.2 ± 39.5 min of walking, 50.8 ± 28.5 min of standing and 21.7 ± 29.1 min of physically demanding tasks during a normal workday.

Standing time per day during office hours increased about 40 min per day from T_1_ to T_3_ (*p* < 0.01).

During baseline, one participant reported that he/she had a height-adjustable desk available, at T_2_ and T_3_ eleven (91.7%) participants had one available. During T_2_, five participants (45.5%) and during T_3_ four participants (36.4%), reported regularly using the height-adjustable function of their desk (Table 3).

## 4. Discussion

The overall aim of this pilot study was to analyze whether the relocation of a small-size office to an active design office building incorporating biophilic design elements and various standing possibilities would change workplace sitting time during working hours and self-reported habit strength concerning uninterrupted sitting and PA during work. The main outcome was that desk-based workers reported a sustainable increase in their standing time of 40 min per working day.

The reported baseline minutes per working day for sitting, standing, walking and physically demanding tasks were slightly higher than previously reported by a German representative sample [46], which could be explained by the higher proportion of full-time workers in the present study. The compensation of sitting time with standing within the present study seems to be in line with the meta-analyses of the effects of activity-permissive workstations [47]. It further supports the results of other natural experiments describing the effects of moving to an active design office on sitting and standing time [27,28]. However, the increase in standing time in our study is higher than in the studies of Jancey et al. [27] and Engelen et al. [28], most probably because in our pilot study the entire staff received sit-and-stand desks. Regarding the rather low regular use of the height-adjustable desks at individual workstations during time point T_3_ (36.4%), the increase in standing time did not seem to be induced solely by the height-adjustable desks. Potentially, sitting time also seemed to be substituted by standing at other office locations, such as in meeting rooms and common spaces, which featured counter desks at standing height. Furthermore, the work tasks of the employees may have involved a change of location within the office due to the redesigned office´s philosophy of task-specific workstations presenting special meeting areas and common areas with standing possibilities. The structure of the redesigned office aimed to invite the office employees to change locations from time to time and consequently frequently use the standing options. While it must be noted that the increase in movement within the office was not statistically significant, participants described an increase of walking time during office hours of about 15 min per day. The trend of an increase in walking time is comparable with the increase of steps during working hours (1686 steps) found in the study by Jancey and colleagues [27]. In contrast, Engelen and colleagues found no increase in walking time during office hours [28]. The increasing trend in walking time in the present study may be due to the gain in floor space in the new building (old building = 536 m^2^, new office = 650 m^2^), increasing the walking distance from the desk to common rooms and the toilet area for example. 

Future studies that investigate the effects of task-specific working spaces, common areas with standing possibilities, standing meeting rooms, etc. on sitting time, sitting time breaks, standing and walking time, without providing sit-and-stand workstations for the entire staff could be of potential interest. 

Our data did not support the assumption that environmental changes alter the habit of prolonged uninterrupted sitting in the workplace setting [30,33,34]. However, our data showed a reducing trend for the habit strength of “uninterrupted sitting during work”, and meanwhile, the new desired and intended habit of “physical activity during work” strengthened almost similarly. The small, non-significant trend for habit strength in this study could be due to the relatively short period of observation, given that habit changes need time. Therefore, it seems promising for future studies in this field to consider habit strength changes in the long-term, and this could be applied as a meaningful measure to determine the success of replacing unwanted with wanted behavior in interventions, by decreasing prolonged uninterrupted occupational sitting and increasing ambulatory PA in the occupational setting, respectively. 

Surprisingly, the level of satisfaction with the office environment persisted and is in contrast with the results of Engelen and colleagues [28]. One explanation for the lack of an overall increase in satisfaction could be due to sun shields which were missing up to the time-points of T_2_ and T_3_, and which were reported by the employers and troubled employees during warm and sunny weather, because of rising temperatures inside the office (sun shields were installed after time-point T_3_). This assumption is cautiously supported by the negative trend for “office thermal comfort”. 

Perceived job performance and work engagement did not alter during the longitudinal view of the relocation of the office and sustained an average level compared to norm scores [48]. This unchanged level of work engagement indicates that work engagement seemed to be unaffected by the increase in standing time during office hours. 

Overall, the possible shift from sitting to more standing time of employees in the office workplace through the introduction of active and biophilic design approaches, without altering job performance and job satisfaction, must be investigated in future larger-scale studies to examine generalizability. Specifically, these results need to be replicated for a wider range of organizations and adaptions of the workplace, in particular to highlight which particular changes in the office space trigger which changes in workplace behavior or outcomes at work.

The strength of the present pilot study is its longitudinal view of the pre-post experimental design of the relocation of an office to an “active” biophilic designed office, including two measurement points after relocation to identify sustainability. With the present study design, participants serve as their own control group. The holistic methodological study approach in gathering information about sitting time, habit strength, environmental satisfaction, and job performance is a strength of this pilot study. The main limitation of this study is the small sample size that dampens generalizability. Larger studies with different workforces that investigate restructuring workspaces into active and biophilic design buildings are warranted. Additionally, there was a >50% dropout rate of participants during data collection (between T_1_ to T_3_). Participants who completed the study may be more likely to be more compliant with the intervention than those who dropped out, which might introduce bias. A further limitation is that the data were obtained based on subjective self-perceptions of sitting, standing, walking and doing physically demanding tasks in the workplace setting. Self-reporting of sitting is prone to potential bias via misclassifications or social desirability and could have been controlled through objective measures [49]. Nevertheless, the self-report scales have been used in such contexts before [28] and report substantial repeatability and validity [38,39,40]. Furthermore, we cannot assign the alteration of the different dependent variables to a specific design approach because diverse approaches were applied here, i.e., biophilic and active design. 

## 5. Conclusions

The results of this study suggest that relocating or altering offices to become more active and using biophilic design could be promising approaches in increasing standing time. Future larger scale studies are warranted to investigate the influence of office design on sitting time and ambulatory activity during working hours in more depth, as well as on habit strength concerning prolonged sitting and PA during working hours.

## Figures and Tables

**Table 1:** Characteristics of the old and new offices.

Characteristic	Old Office	New Office
Size in qm^2^	536 qm^2^ (ground floor: 363 qm^2^ basement floor: 173 qm^2^)	650 qm^2^
Number of employees	23	23
Work stations (height-adjustable work stations)	21 (4)	22 (22)
Meeting areas	Three conference rooms (a) table with three lounge chairs (b) classic conference room with four chairs (c) showroom with conference table	Five conference rooms (a) table with eight lounge chairs (b) classic conference room with 12 chairs (c) theatre with benches (d) standing conference table with three counter chairs (e) table with three chairs
Common space	Classic kitchen (first floor) with a table and six chairs	Two public spaces (a) Ground floor: 2 counter tables with counter chairs (b) Basement floor: kitchen with classic table with eight chairs and counter table with counter chairs
Floors	2	2
Stairs	Internal stairs (connecting ground floor and basement)	Internal stairs (connecting ground floor and basement) plus external staircase
Design approach	Classic open space	Physically active and biophilic design
ActiveOffice® features	-	Wall bars, rings, punch pad, floor surface arrangements to jump over boxes, different standing and sitting options, lying surfaces, etc.

**Table 2:** Means, standard deviations (SDs) and statistical analyses of outcome variables at baseline (pre-location (T_1_), three months after relocation (T_2_), and seven months after relocation (T_3_)).

Item	T_1_	T_2_	T_3_	*p*	F	part. η^2^	*n*
Total and domain-specific sitting time during weekdays							
Transport-related sitting time (min per day)	75.0 ± 33.9	77.5 ± 34.3	90.8 ± 79.9	0.80	0.23	0.04	12
Work-related sitting time (min per day)	422.5 ± 74.0	370.0 ± 66.9	350.0 ± 96.0	0.03	4.88	0.49	12
TV-related sitting time (min per day)	75.0 ± 55.0	87.5 ± 60.6	77.5 ± 55.0	0.41	0.98	0.16	12
Leisure-computer-related sitting time (min per day)	47.5 ± 43.3	72.5 ± 60.6	62.5 ± 37.2	0.51	0.73	0.13	12
Leisure-related sitting time (min per day)	101.3 ± 63.0	77.5 ± 43.3	87.5 ± 68.2	0.41	0.98	0.16	12
Total sitting time (min per day)	721.3 ± 96.1	685.0 ± 87.3	668.3 ± 170.8	0.34	1.21	0.20	12
Sitting time and PA in the office environment							
Min/workday of sitting	364.2 ± 102.5	294.6 ± 100.3	288.6 ± 64.2	0.07	3.68	0.45	11
Min/workday of walking	70.2 ± 39.5	87.0 ± 47.5	84.0 ± 33.9	0.33	1.26	0.22	11
Min/workday of standing	50.8 ± 28.5	104.1 ± 103.9	91.9 ± 36.2	0.01 ** T_1_ vs. T_3_	9.25	0.67	11
Min/workday of physically demanding tasks	21.7 ± 29.1	20.0 ± 23.5	29.8 ± 35.8	0.76	0.29	0.05	12
Self-reported breaks in sitting time in the workplace setting							
Mean number of breaks of sitting time during one hour	3.8 ± 2.2	3.4 ± 1.6	4.2 ± 1.7	0.31	1.31	0.21	12
Self-reported habit strength							
Habit strength ’uninterrupted sitting during work’	4.9 ± 1.4	4.4 ± 1.6	4.4 ± 1.7	0.11	2.80	0.36	12
Habit strength ’physical activity during work’	4.0 ± 1.4	4.1 ± 1.5	4.5 ± 1.8	0.67	0.42	0.08	12
Satisfaction with the office environment							
Office acoustics	4.9 ± 1.7	4.2 ± 1.9	4.4 ± 1.5	0.69	0.39	0.07	12
Office indoor air quality	4.3 ± 1.5	3.5 ± 1.8	3.7 ± 1.8	0.52	0.70	0.12	12
Office lighting	3.1 ± 1.7	4.6 ± 1.9	4.3 ± 1.7	0.17	2.15	0.30	12
Office layout	3.1 ± 1.1	3.2 ± 1.6	2.7 ± 1.1	0.07	3.60	0.42	12
Office furnishings	3.1 ± 1.1	2.1 ± 1.1	2.2 ± 1.3	0.09	3.11	0.38	12
Office thermal comfort	3.9 ± 1.7	3.6 ± 1.8	4.2 ± 1.9	0.38	1.06	0.18	12
Overall satisfaction	3.3 ± 1.4	3.3 ± 1.6	2.8 ± 1.6	0.49	0.78	0.13	12
Work engagement							
Overall work engagement	3.9 ± 0.8	3.7 ± 0.8	4.0 ± 1.2	0.43	0.93	0.16	12
Job performance							
Task Performance	4.6 ± 0.4	4.6 ± 0.4	4.3 ± 1.1	0.53	0.67	0.12	12
Contextual Performance	4.4 ± 0.4	4.1 ± 0.6	4.1 ± 1.0	0.27	1.50	0.23	12

** *p* < 0.01.

**Table 3:** Availability of height-adjustable desks and use of height-adjustable desks at baseline (pre-location (T_1_), three months after relocation (T_2_), and seven months after relocation (T_3_)).

Item	Answer	T_1_	T_2_	T_3_
Availability of height-adjustable desk	Yes	1 (8.3%)	11 (91.7%)	11 (91.7%)
	No	11 (91.7%)	1 (8.3%)	1 (8.3%)
Regular use of height-adjustable desk	Yes, regularly	0 (0.0%)	5 (45.5%)	4 (36.4%)
	No, not regularly	1 (100.0%)	6 (54.5%)	7 (63.6%)

## Data Availability

The datasets analyzed during the current study are available from the corresponding author on request.

## Abbreviations

OSPAQ	Occupational Sitting and Physical Activity Questionnaire
PA	physical activity
